# Altered Metabolic Activity and Morphology of Lower Limb Muscles 1–2 Years Following Anterior Cruciate Ligament Reconstruction

**DOI:** 10.1111/sms.70079

**Published:** 2025-05-24

**Authors:** Benjamin Dutaillis, Tyler Collings, Philip Bellinger, Ryan G. Timmins, Benjamin Kennedy, Matthew N. Bourne

**Affiliations:** ^1^ School of Health Sciences and Social Work Griffith University, Gold Coast Campus Gold Coast Australia; ^2^ Australian Centre for Precision Health and Technology (PRECISE) Griffith University, Gold Coast Campus Gold Coast Australia; ^3^ Griffith Sport Science Griffith University, Gold Coast Campus Gold Coast Australia; ^4^ School of Behavioural and Health Sciences Australian Catholic University Brisbane Australia; ^5^ The Sports Performance, Recovery, Injury and New Technologies (SPRINT) Research Centre Brisbane Australia; ^6^ Mermaid Beach Radiology Gold Coast Australia

**Keywords:** biomechanics, knee injury, vertical jump‐landing

## Abstract

The purpose of this study was to explore between‐limb differences in gluteal, quadriceps, hamstring, and triceps surae muscle size and activity during a repeated vertical jump‐landing task in individuals with a history of unilateral anterior cruciate ligament reconstruction (ACLR). Twelve recreationally active participants with a unilateral history of ACLR involving a semitendinosus autograft (1–2.5 years post‐surgery) underwent functional magnetic resonance imaging (fMRI) before and immediately after 60 vertical jumps. Exercise‐induced increases in transverse (T2) relaxation times and resting muscle volumes were measured in 12 lower limb muscles. Linear mixed models were used to explore differences between the ACLR and uninjured contralateral limb, as well as their relationship with vertical jump performance. Reconstructed limbs displayed smaller muscle volumes in vastus medialis (*p* = 0.028), rectus femoris (*p* = 0.019), semitendinosus (*p* < 0.001), and gastrocnemius lateral head (*p* = 0.028) than homonymous muscles in the uninjured contralateral limb. Limbs with a history of ACLR also exhibited smaller percentage changes in T2 relaxation time for semitendinosus (*p* < 0.001), semimembranosus (*p* = 0.002), lateral gastrocnemius (*p* = 0.014), and soleus (*p* = 0.008), while the vastus lateralis displayed a greater increase (*p* = 0.001) than uninjured contralateral limbs. Between‐limb asymmetry in quadriceps muscle volume and activity was associated with between‐limb asymmetry in vertical jump‐landing impulse (r^2^ = 0.30–0.34). The findings from this study may help to inform the design of exercise interventions aimed at restoring lower limb function and reducing reinjury risk in individuals with a history of ACLR.

## 1 Introduction

Anterior cruciate ligament (ACL) ruptures are serious knee injuries that are increasingly common in field and court sports [1]. ACL reconstruction (ACLR) remains the primary treatment option to restore passive knee joint stability [2]. However, despite surgery and rehabilitation, nearly half of all athletes do not return to competitive sport [3], and 20%–25% sustain a secondary ACL injury [4]. This highlights the potential limitations of conventional ACLR rehabilitation practices and suggests that more is to be learned about the mechanisms contributing to poor post‐surgical outcomes.

Long‐term deficits in vertical jump performance have been reported when comparing reconstructed limbs to uninjured contralateral limbs and the limbs of healthy controls [5]. Prior work has demonstrated that limbs with ACLR exhibit decreased knee joint power and work [6, 7, 8], as well as altered lower limb muscle contributions—such as reduced force from soleus and rectus femoris [8]—when performing vertical jumps. Consequently, contemporary clinical practice guidelines recommend assessing between‐limb asymmetry in force‐plate derived measures of vertical jump performance as part of return to play criteria [9]. However, limited work has explored between limb differences in lower limb muscle activation during vertical jumping in individuals with a history of ACLR. A recent systematic review reported reductions in normalized surface electromyography (sEMG) activity of the quadriceps and increased activity of the hamstrings in previously injured limbs during vertical jumping [10]. However, sEMG has limitations, such as susceptibility to cross‐talk [11], difficulty distinguishing between closely approximated muscles (e.g., individual gluteal muscles or medial and lateral hamstrings) [11], and the coefficient of variation for repeated measures can be as high as 23% [12]. Furthermore, few studies have explored the activation patterns of muscles outside of the quadriceps and hamstrings during vertical jumping in individuals with a history of ACLR, leaving a gap in our understanding of overall muscle activity during dynamic movements.

Lower limb muscle atrophy is another well‐known consequence of ACLR [13]. Reconstructed limbs typically display deficits in quadriceps muscle volume, which are most pronounced during the first 6 months of recovery [13]. Moreover, a significant proportion of ACLR procedures involve semitendinosus autografts, which are associated with chronic reductions in semitendinosus volume [13]. Deficits in quadriceps [14] and hamstring [15] muscle volumes reduce knee extension and flexion torque‐producing capacity, respectively, which may impact lower limb muscle function during high‐speed, athletic tasks. However, limited work has examined the impact of ACLR on the morphology of other major lower limb muscles [13, 16]. Further, no studies have concurrently explored between‐limb differences in lower limb muscle volumes and muscle activity during a vertical jump‐landing task.

Muscle functional magnetic resonance imaging (fMRI) offers a high‐resolution method to assess muscle size and quantify muscle activity during exercise [17]. This imaging technique detects exercise‐induced increases in the transverse (T2) relaxation time of tissue water, which increase in proportion to exercise intensity [18] and demonstrate a consistent relationship with integrated sEMG measures of muscle activity [18]. However, unlike sEMG, fMRI can map these T2 changes in cross‐sectional images of muscles along their length, providing significantly greater spatial resolution. Additionally, exercise‐induced T2 shifts demonstrate excellent reproducibility (intra‐class correlation coefficients = 0.87–0.94) [19], which is an important advantage over sEMG methods. Functional MRI has recently been used to examine between‐limb differences in hamstring muscle activity during knee flexor exercises following ACLR [20, 21, 22, 23]. However, to the authors' knowledge, it has never been applied to explore lower limb muscle activity in more dynamic athletic tasks, such as vertical jumping.

An improved understanding of lower limb muscle size and activity during jump‐landing tasks in individuals with a history of ACLR could reveal patterns associated with reduced performance and may have implications for the design of more targeted rehabilitation or return to sport strategies. Therefore, the primary aims of this study were to:

1. Examine the between‐limb differences in lower limb muscle volumes and muscle activity during a repeated vertical jump‐landing task in athletes with a unilateral history of ACLR, and
2. Explore the extent to which between‐limb asymmetry in muscle volume and activity is associated with between‐limb asymmetry in vertical jump performance.

## 2 Methods

### 2.1 Participants

Twelve recreationally active participants (*n* = 5 male, 25.0 ± 4.6 years, 181.8 ± 4.5 cm, 80.2 ± 13.3 kg and *n* = 7 female, 20.4 ± 1.0 years, 152.3 ± 40.5 cm, 69.4 ± 9.3 kg) who had undergone primary, unilateral ACLR involving a semitendinosus autograft were recruited for this study. Using G*power (version 3.1.9.7), a sample size of *n* = 12 was determined appropriate to achieve a power of 0.8 (*α* = 0.05), based on an anticipated effect size of *d* = 0.8, estimated from prior literature [21, 22]. All participants had undergone standard care rehabilitation under the guidance of a qualified physiotherapist and reported undertaking high‐intensity resistance and plyometric training aimed at restoring lower limb function throughout recovery. Inclusion criteria were: (i) aged 18–35 years, (ii) history of unilateral ACLR involving an autograft from the ipsilateral semitendinosus, and (iii) cleared to return to play from their treating surgeon. Exclusion criteria involved: (i) contraindications to MRI, (ii) prior or current major lower limb or lower back injuries, and (iii) history of neurological or cardiometabolic disorders. The mean time since surgery was 72.0 weeks (range 49.7–136.0). Prior to testing, all participants completed a cardiovascular screening questionnaire to ensure it was safe for them to exercise and a standardized MRI questionnaire to ensure it was safe for them to enter the magnetic field. All participants provided written informed consent to participate in the study, which was approved by the Griffith University Human Research Ethics Committee (2023/684).

### 2.2 Study Design

This cross‐sectional study involved a single testing session. Upon arrival at the imaging facility, participants were seated for a minimum of 15 min to minimize intramuscular fluid shifts. Subsequently, participants underwent fMRI of their lower limbs before and immediately after a repeat jumping protocol.

### 2.3 Magnetic Resonance Imaging

All imaging was performed using a 3‐Tesla MRI system (Philips 3T Elition X). Participants were positioned supine in the magnetic bore with their hips in neutral and knees fully extended, with anterior body coils placed over their anterior hips, thighs, and shank. Scans of both lower limbs began just superior to the iliac crest and finished inferior to the lateral malleolus. Prior to exercise, participants underwent two imaging sequences to generate axial T1‐weighted 3D mDixon and T2‐weighted images. T2‐weighted imaging was repeated immediately after exercise. T2‐weighted mapping images were acquired using a multi‐echo, fast spin echo sequence, with a total scan time of 465 s. A localiser scan was completed prior to the T2‐weighted sequence to standardize the field of view and align the pre‐ and post‐exercise images. Scan sequence details can be found in Table 1. To ensure no abnormal fluid shifts were present during imaging, participants were asked to refrain from lower limb exercise for a minimum of 48 h prior to testing.

**TABLE 1:** Magnetic resonance imaging scan sequence details.

	T2‐weighted	3D mDixon
Slice thickness	10 mm	2 mm
Slice gap	10 mm	0 mm
Field of view	270 × 381 mm	350 × 448 mm
Relaxation time, TR	3100 ms	5 ms
Echo time, TE	8, 16, 24, 32, 40, 48, 56, 64 ms	1.94, 3.4 ms
Number of echoes	8	2
Voxel size	Acquired 2 × 2 × 10 mm Recon 0.5 × 0.5 × 10 mm	Acquired 1 × 1 × 2 mm Recon 0.44 × 0.44 × 2 mm
Stations	3	4
Slices per station	15	136
Acquisition time	155 s	30 s

### 2.4 Vertical Jumping Protocol

All participants were familiar with vertical jump testing using portable force‐plates, having undergone testing regularly by their treating clinicians during rehabilitation. Participants completed double leg countermovement‐rebound jumps [24], whereby they executed a maximal countermovement jump and upon landing immediately performed a second maximal countermovement jump. Participants were instructed to maintain their hands on their hips and jump “as high and fast as possible”, with a self‐selected depth, aiming to maximize height and minimize ground contact time for all repetitions. Strong verbal encouragement was provided by the investigators to encourage maximum effort during each jump. Participants completed 30 repetitions (total of 60 jumps), with 10 s rest between repetitions to ensure a detectable change in T2 relaxation time, while minimizing the effects of fatigue [25]. Using dual‐portable force‐plates (ForceDecks; VALD, Brisbane), ground reaction forces (GRFs) were recorded separately for each limb. Prior to the jumping protocol, the force‐plates were zeroed and participants were weighed, as per manufacturer procedures. The repeated jumping protocol took a total of 385 s to complete. All participants walked (~5 m) to the scanner immediately following cessation of the jumping protocol, and post‐exercise T2‐weighted imaging began within 155 ± 11 s. The mean positive impulse (i.e., impulse greater than body mass) was calculated for each limb during the countermovement rebound jump via summing the eccentric and concentric phase impulses extracted from ForceDecks (Vald, Brisbane), and averaging across all trials.

### 2.5 Data Analysis

T2 relaxation time and muscle volumes were analyzed for 12 major lower limb muscles: gluteus medius, gluteus maximus, rectus femoris, vastus lateralis, vastus medialis, vastus intermedius, semitendinosus, semimembranosus, biceps femoris long head, gastrocnemius lateral and medial heads, and soleus.

#### 2.5.1 T2 Relaxation Time

To examine the extent of muscle activity during vertical jumping in previously injured and uninjured contralateral limbs, the T2 relaxation time of the aforementioned muscles was measured in the pre‐ and post‐exercise scans. T2‐weighted images were imported into Horos v3.3.6 (The Horos Project, Annapolis, MD, USA). T2 maps were reconstructed via fitting signal intensity values of each echo (8, 16, 24, 32, 40, 48, 56, and 64 ms) to a mono‐exponential decay curve. Within each muscle, regions of interest (ROI) were placed on the T2 maps in five separate axial slices along the mid‐section of the muscle belly, corresponding to ~50% of each muscle's length (estimated from the first and last appearance of each muscle of interest on axial slices), and the two slices immediately cranial and caudal [22]. The size of ROIs ranged from 0.5 to 10 cm^2^ depending on the CSA of the muscle and the amount of homogenous tissue in each slice. Care was taken to align ROIs in the pre‐ and post‐exercise slices while avoiding blood vessels, nervous tissue, fatty tissue, and dielectric artifact. Where a homogenous region of muscle tissue was not able to be segmented, this slice was removed from the analysis. The final inclusion counts (out of a possible 120 slices across all participants) were: gluteus medius = 120, gluteus maximus = 120, vastus lateralis = 120, vastus medialis = 120, vastus intermedius = 120, semimembranosus = 120, biceps femoris = 120, gastrocnemius medial head = 118, gastrocnemius lateral head = 112, soleus = 112, semitendinosus = 74, and rectus femoris = 60. Calculation of change in muscle T2‐relaxation time has previously demonstrated excellent interrater reliability of (ICCs = 0.87–0.94) [19].

The percentage change in T2 relaxation time from pre‐ to post‐exercise for each muscle, in each slice, was calculated using the following formula [22]:

$$\begin{array}{c} \%\;\text{change in T2}=\left(\text{T2 post}‐\text{exercise}–\text{T2}\;\mathrm{pre}‐\text{exercise}\right)\\ \;/\text{T2}\;\mathrm{pre}‐\text{exercise}\times 100 \end{array}$$

#### 2.5.2 Muscle Volumes

Muscle volumes were determined for the previously injured and uninjured contralateral limbs from T1‐weighted 3D mDixon images. A commercially available deep convolutional neural network methodology (Springbok Analytics, Virginia, USA) [26] was used to automatically segment muscle structures bilaterally. Segmentations were then manually reviewed and edited by trained segmentation engineers who were blinded to participant identity and the previously injured limb. Muscle volumes were quantified by summing the voxels per segment and multiplying by the voxel's volume. Muscle volumes derived from this method display excellent reliability (dice score = 0.87–0.97) [26].

### 2.6 Statistical Analysis

Statistical analysis was performed in R Studio (R version 4.2.0). Between‐limb differences in (1) the percentage change in T2 relaxation time from pre‐ to post‐exercise, and (2) muscle volumes were assessed using linear mixed effects models via the “lme4” and “LmerTest” packages [27]. Separate models were fitted for T2 relaxation time shifts and volumes of each lower limb muscle (i.e., gluteus medius, gluteus maximus, rectus femoris, vastus lateralis, vastus medialis, vastus intermedius, semitendinosus, semimembranosus, biceps femoris long head, gastrocnemius lateral and medial heads, and soleus). Models included limb as a fixed effect, sex and time since surgery as fixed effect covariates, and participant as a random effect (random intercepts). Statistical significance was set at *α* = 0.05. To maintain a false discovery rate of 5%, a correction was applied to the *p*‐values for the muscle volume and T2 relaxation time variables separately, using the Benjamini and Hochberg method in the “rstatix” package. Comparisons were reported as marginal mean differences with 95% confidence intervals (CIs) (emmeans package). Cohen's *d* was reported as a measure of the standardized effect size, with the levels of effect interpreted as small (*d* = 0.20), medium (*d* = 0.50) or large (*d* = 0.80) [28]. Between‐limb difference in mean positive impulse was reported descriptively as a measure of jump performance.

Univariate linear regression was used to explore the extent to which between‐limb asymmetry in muscle volumes and percentage changes in T2 relaxation times were associated with between‐limb asymmetry in mean positive impulse. Models were built for each measure of muscle volume asymmetry and T2 relaxation time shift asymmetry as predictor variables, and mean total impulse asymmetry as the response variable. Between‐limb asymmetry was calculated for muscle volumes, T2 shifts, and mean total impulse using the following formulas:

*Muscle volumes*

$$\left(\text{uninjured}-\text{ACLR}\right)/\text{uninjured}\times 100$$

*T2 shifts and mean total impulse*

$$\left(\text{uninjured}–\text{ACLR}\right)/\left(\text{uninjured}+\text{ACLR}\right)\times 100$$

## 3 Results

**FIGURE 1:**
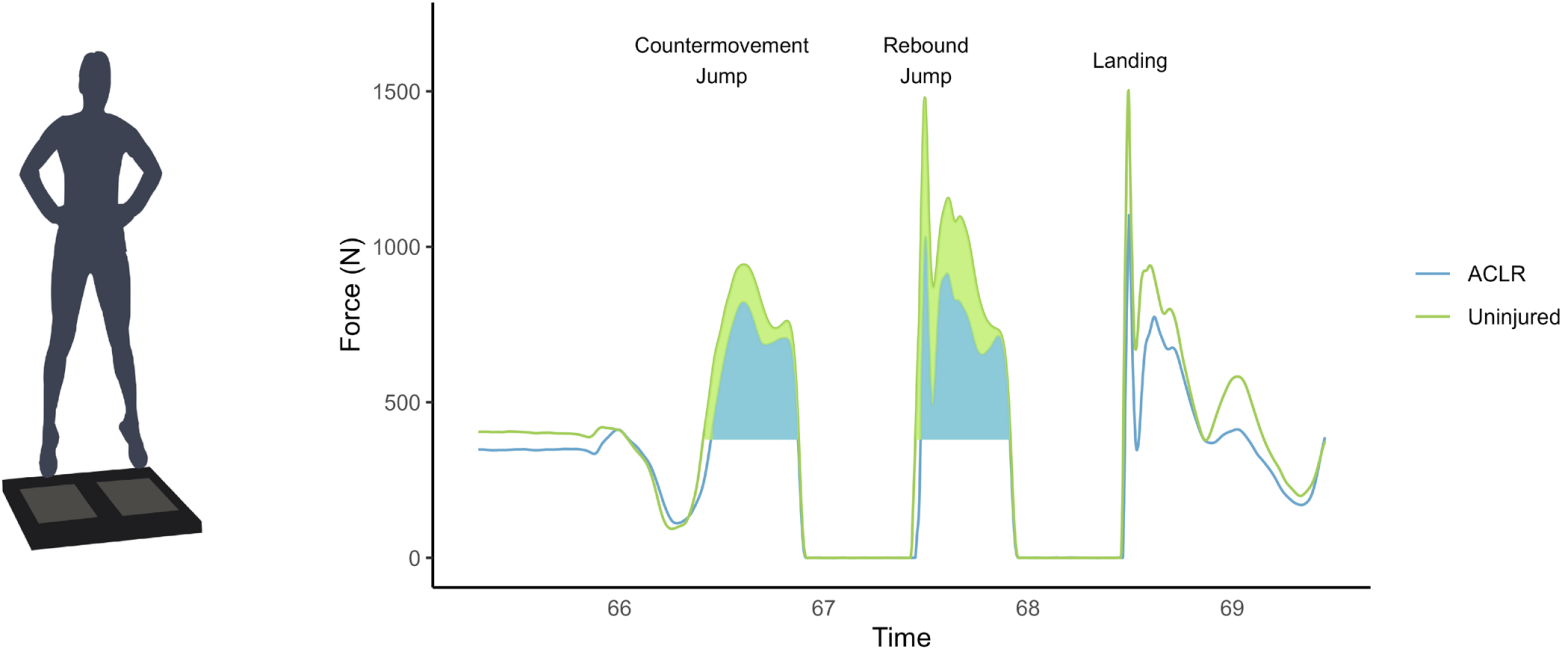
Example of a countermovement rebound jump ground reaction force (GRF) waveform in the anterior cruciate ligament reconstructed (ACLR) (blue) and uninjured contralateral (green) limbs. Shaded areas show the positive impulse (i.e., area under the curve greater than body mass) during the countermovement rebound jump in the ACLR and uninjured contralateral limbs, respectively.

All participants completed the vertical jump protocol, and none reported any pain or discomfort before or immediately after exercise. Limbs with a history of ACLR displayed lower mean positive impulse during the countermovement rebound jump (mean difference = −26.6 Ns, 95% CI = 6.1–47.2, *d* = −1.1) than uninjured contralateral limbs (Figure 1). Jump heights showed a small increase of less than 2 cm from first to last jump (coefficient = 0.05 cm).

### 3.1 Between‐Limb Differences in T2 Relaxation Time Changes

**FIGURE 2:**
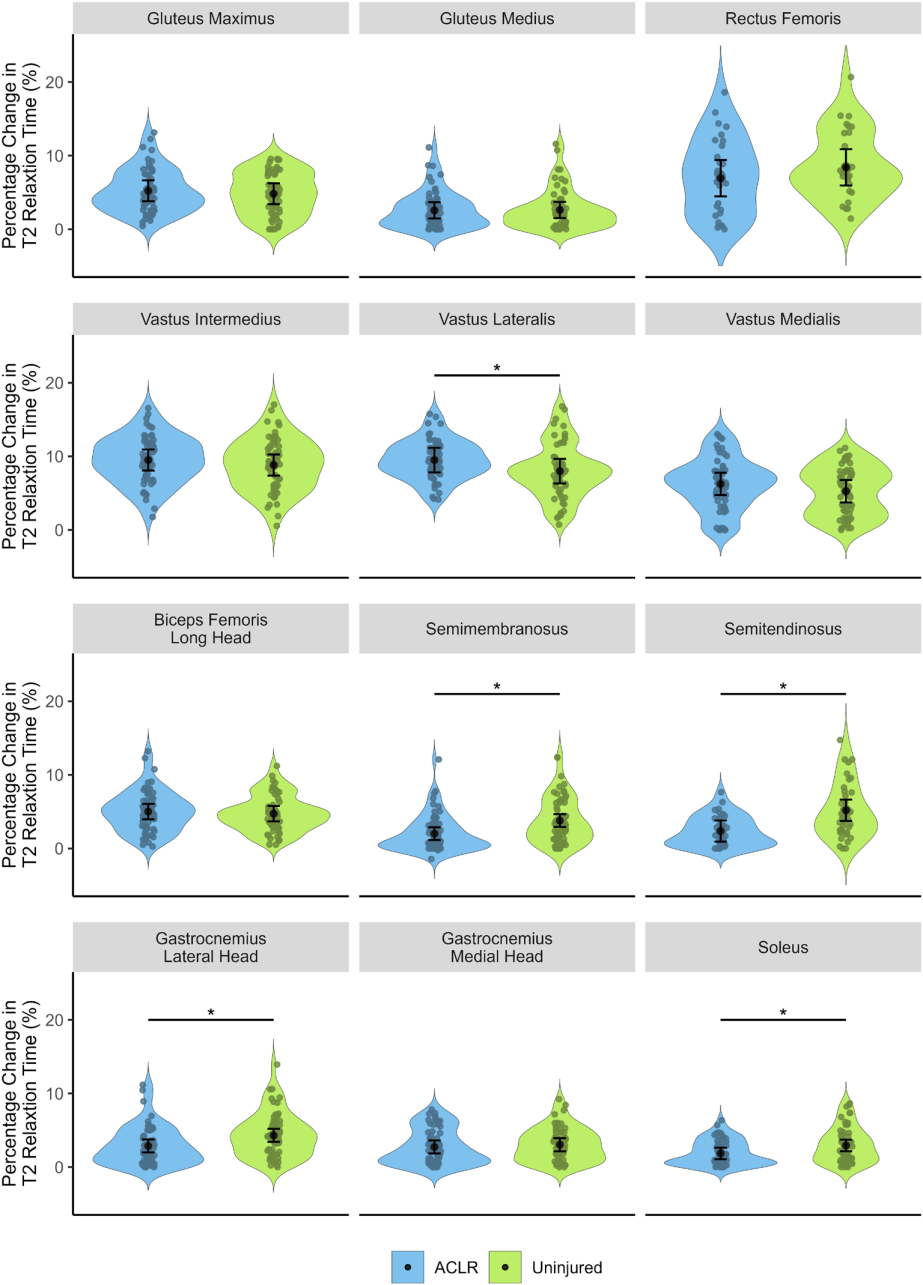
Percentage change in T2 relaxation time after vertical jumping in limbs with a prior anterior cruciate ligament reconstruction (ACLR) (blue) and uninjured contralateral limbs (green). Small colored circles represent individual data points, while black circles and error bars show marginal means (95% confidence intervals). *Denotes significant between‐limb difference (*p* < 0.05).

**FIGURE 3:**
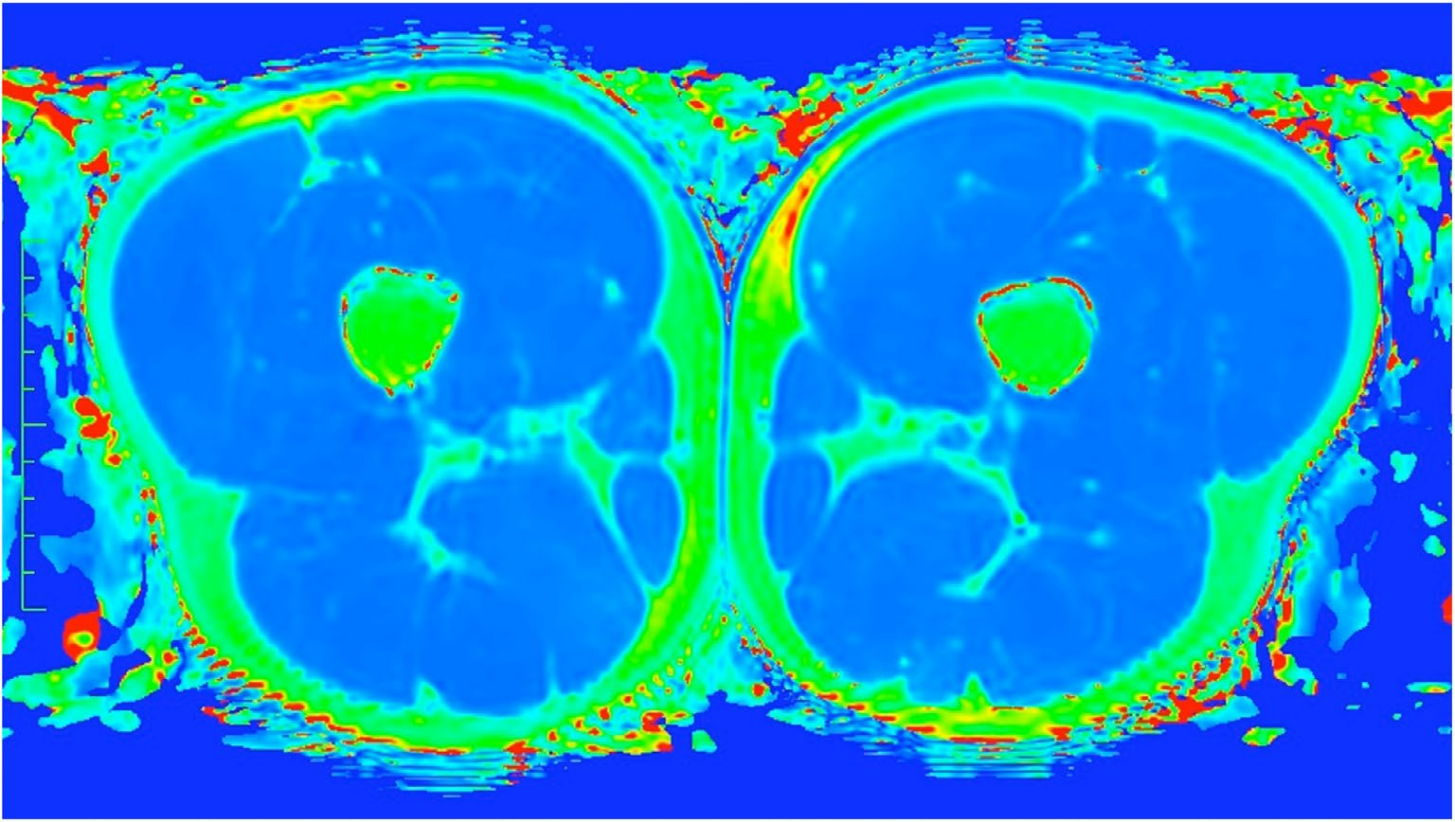
T2‐weighted map of a mid‐thigh axial slice showing the previously injured (left) and uninjured contralateral (right) limbs. The right side of the image corresponds to the participant's left side as per radiology convention. T2 relaxation time was measured via placing regions of interest inside a homogenous region of muscle tissue, seen here in dark blue.

Reconstructed limbs displayed a significantly lower percentage change in T2 relaxation time after jumping for semitendinosus (mean difference = −2.8%, 95% CI = −1.6 to −4.0, *d* = −1.1, *p* < 0.001), semimembranosus (mean difference = −1.8%, 95% CI = −0.8 to −2.7, *d* = −0.7, *p* = 0.002), lateral gastrocnemius (mean difference = −1.4%, 95% CI = −0.4 to −2.4, *d* = −0.5, *p* = 0.014), and soleus (mean difference = −1.1%, 95% CI = −0.4 to −1.8, *d* = −0.6, *p* = 0.008) compared to homonymous muscles in the uninjured contralateral limb (Figures 2 and 3). In contrast, the vastus lateralis of limbs with a history of ACLR exhibited a greater percentage increase in T2 relaxation time than the uninjured contralateral limb (mean difference = 1.5%, 95% CI = 2.3–0.8, *d* = 0.7, *p* = 0.001). No other significant between‐limb differences in exercise‐induced T2 relaxation time changes were observed for any other muscle.

### 3.2 Between‐Limb Differences in Muscle Volumes

**FIGURE 4:**
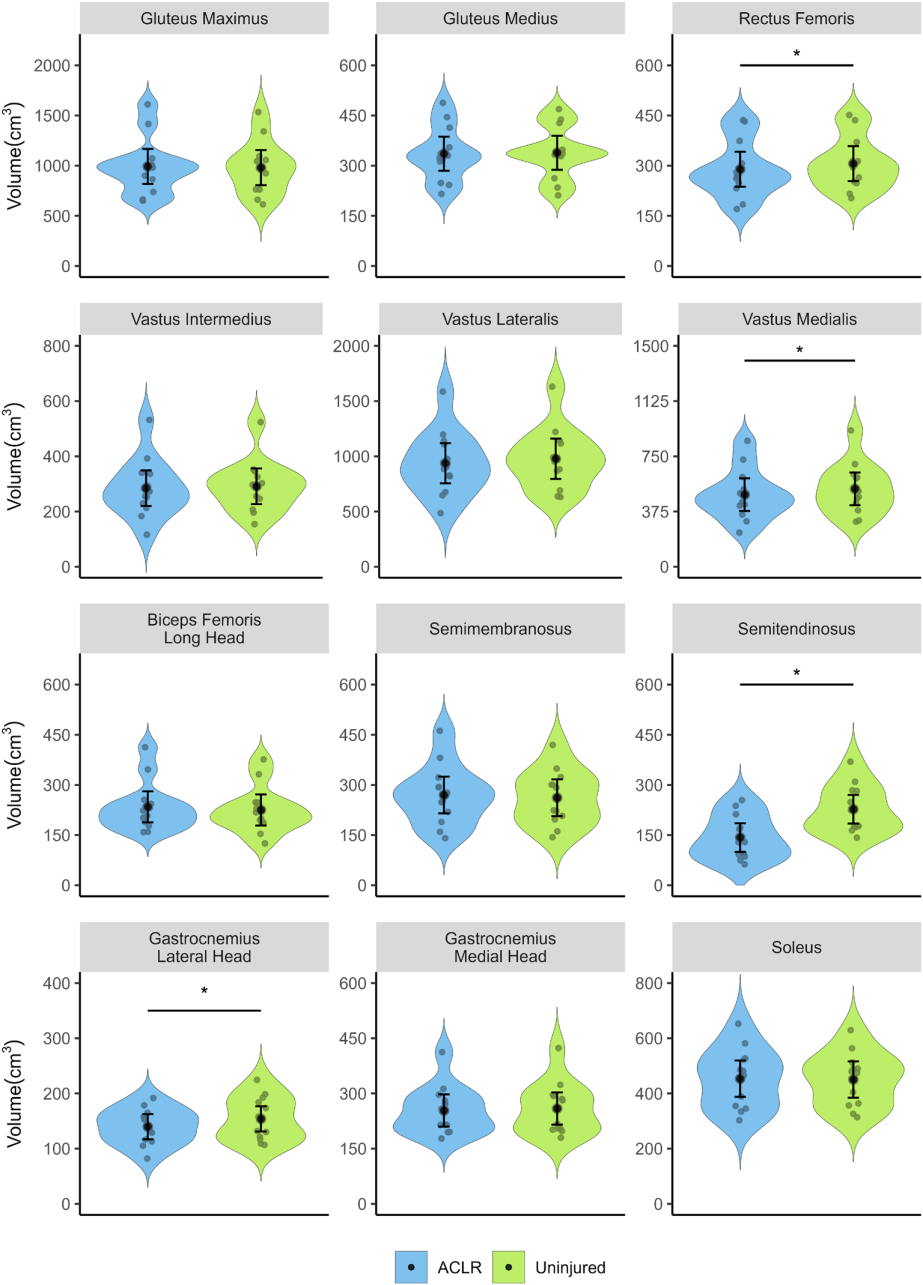
Muscle volumes for limbs with a prior anterior cruciate ligament reconstruction (ACLR) (blue) and uninjured contralateral limbs (green). Small colored circles represent individual data points, while black circles and error bars shows marginal means (95% confidence intervals). *Denotes significant between‐limb difference (*p* < 0.05).

**FIGURE 5:**
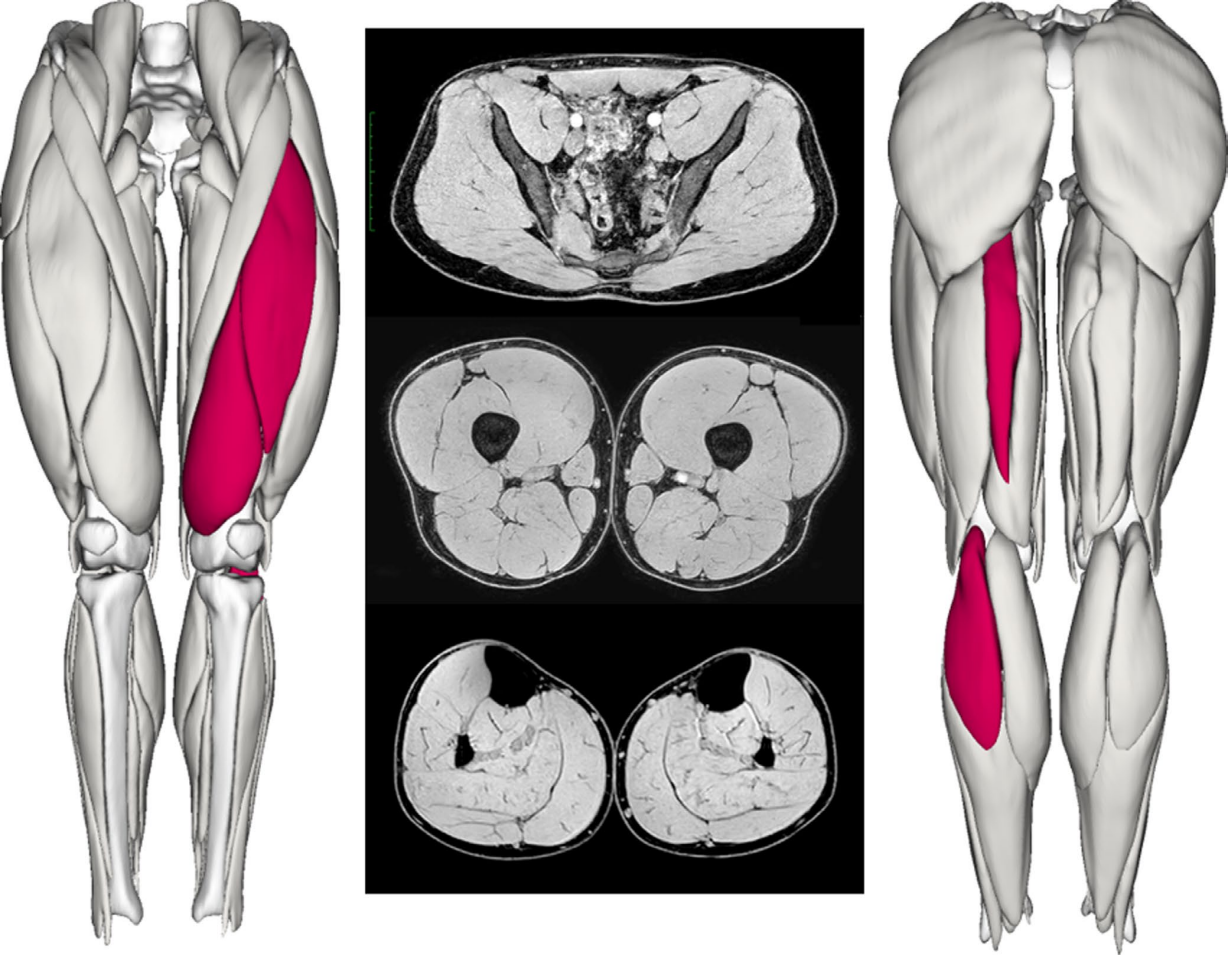
Middle panel shows representative T1‐weighted mDixon axial slices from mid‐gluteal (top), mid‐thigh (middle), and mid‐shank (bottom). Left and right panels show the reconstructed 3D renders of lower limb muscle volumes, which were calculated from contiguous axial slices across the length of each muscle belly. Red coloring highlights muscles (vastus medialis, rectus femoris, semitendinosus and gastrocnemius lateral head) that were significantly different (*p* < 0.05) in the anterior cruciate ligament reconstructed limb compared to the uninjured contralateral limb.

Reconstructed limbs displayed smaller vastus medialis (mean difference = −38.72 cm^3^, 95% CI = −14.14 to −63.30, *d* = −1.3, *p* = 0.028), rectus femoris (mean difference = −16.79 cm^3^, 95% CI = −7.66 to −25.9, *d* = −1.5, *p* = 0.019), semitendinosus (mean differences = −84.77 cm^3^, 95% CI = −65.25 to −104.29, *d* = −3.6, *p* < 0.001), and gastrocnemius lateral head volumes (mean difference = −14.17 cm^3^, 95% CI = −4.99 to −23.35, *d* = −1.3, *p* = 0.028) than uninjured contralateral limbs (Figures 4 and 5). No other significant between‐limb differences in volume were observed for any other muscle.

### 3.3 Association Between Muscle Size, Activity and Countermovement Rebound Jump Impulse

Univariate linear regression revealed that between‐limb asymmetry in vastus medialis percentage change in T2 relaxation time (coefficient = −0.04, *r* ^2^ = 0.34, *p* = 0.02), vastus medialis volume (coefficient = 0.22, *r* ^2^ = 0.31, *p* = 0.03), and vastus intermedius volume (coefficient = 0.20, *r* ^2^ = 0.30, *p* = 0.04) was significantly associated with mean positive impulse asymmetry.

## 4 Discussion

This is the first study to use fMRI to explore between‐limb differences in muscle activity during vertical jumping and muscle volumes in athletes with a history of unilateral ACLR. Reconstructed limbs displayed smaller exercise‐induced increases in T2 relaxation time in the medial hamstrings, soleus, and lateral gastrocnemius, and greater increases in the vastus lateralis, compared to homonymous muscles in the uninjured contralateral limb. Additionally, we found evidence for reduced volume of rectus femoris, vastus medialis, semitendinosus, and lateral gastrocnemius in ACLR limbs. These findings highlight significant between‐limb differences in the structure and activity of major lower limb muscle groups that persist even after surgery and rehabilitation.

One of the main findings of this study was that reconstructed limbs displayed greater exercise‐induced T2 relaxation time shifts of the vastus lateralis, alongside smaller vastus medialis and rectus femoris volumes, than uninjured contralateral limbs. The quadriceps are one of the key muscle groups responsible for the acceleration of the body's centre of mass during vertical jumping [29]. Reductions in quadriceps muscle size are likely a consequence of de‐loading and arthrogenic muscle inhibition [30, 31, 32] following injury and surgery, and are known to reduce knee extension strength [14, 31]. Concordantly, lower knee extension strength has been linked to a reduction in knee joint power during vertical jumping [33]. Although the mechanism(s) underpinning greater activity of the vastus lateralis during jumping in limbs with a history of ACLR is unclear, it might conceivably reflect compensation for the reduced force generating capacity of the knee extensors due to rectus femoris and vastus medialis atrophy.

The linear regression models revealed that between‐limb asymmetry in vastus medialis T2 shift and volume, as well as vastus intermedius volume, were associated with impulse asymmetry during vertical jumping. Long‐term asymmetrical jump strategies during double‐leg jumps are known to persist well beyond surgery and rehabilitation [5]. Current clinical practice guidelines advocate for the inclusion of between‐limb asymmetry in eccentric and concentric impulse during vertical jumping as key return‐to‐play criteria [9]. These data suggest the possibility that deficits in quadriceps muscle size and alterations in muscle activity may be important mechanisms underpinning asymmetrical jumping strategy following ACLR.

Observations of semitendinosus atrophy in individuals with a history of ACLR involving a hamstring graft are consistent with previous work [13]. Here, we show that reconstructed limbs also display deficits in the activity of the semitendinosus and semimembranosus during vertical jumping. Prior studies employing fMRI have shown similar deficits in semitendinosus activity of the ACLR limb compared to the uninjured contralateral limb during the performance of the Nordic curl [21] and a prone hamstring curl [22], but not during concentric isokinetic knee flexion [20]. However, this study is the first to show lower levels in repeated vertical jumping. Significant muscle volume loss, retraction, and altered shape of the semitendinosus are known to occur following tendon harvest for ACLR, which reduces muscle‐tendon torque generating capacity even when distal tendon regeneration occurs [15]. However, it is unclear why the semimembranosus displays similarly lower levels of activity in the reconstructed limb. Given the role of the hamstrings in producing posterior shear forces at the knee [34]—and their potential to reduce ACL load during high‐speed tasks like jump‐landing and cutting—lower medial hamstring size and activity during dynamic tasks may contribute to altered joint loading patterns. However, future research is needed to determine the implications of these findings and their potential relationship to ACL reinjury risk [4, 35].

The lateral gastrocnemius displayed significantly lower muscle activity and volume in the reconstructed limb compared to the uninjured contralateral limb. Further, deficits in soleus activity were observed in limbs with a history of ACLR. Gastrocnemius and soleus significantly contribute to vertical jump performance [29]. Prior work has shown a lower contribution from soleus during single‐leg drop jumps in the reconstructed limb compared to the uninjured contralateral limb [8], which paralleled reductions in total ankle work [8]. Deficits in calf muscle size and activation could lower the plantar flexion force producing capacity of the reconstructed limb, negatively influencing jump performance. Soleus has also been found to produce a posterior‐directed shear force at the knee joint [34]. Reductions in soleus muscle activity might conceivably increase the load on the ACL during high‐speed tasks, which could have implications for the risk of secondary injury.

## 5 Limitations

From this cross‐sectional study, we are unable to determine if the differences between reconstructed and uninjured contralateral limbs predated or occurred because of injury. Further, we did not have a control group without a history of ACLR. As such, we do not know if muscle activity in the uninjured contralateral limb was the same or different from limbs with no history of injury. However, uninjured contralateral limbs generally displayed similar lower limb biomechanics as the limbs of healthy athletes with no history of injury during vertical jumping [6, 36]. Time since surgery showed no significant effect on any outcomes, although participants were tested in a relatively narrow window following surgery (1–2.5 years). However, differences in rehabilitation, which were not controlled by the research team, and sports participation before and after surgery may have impacted findings. Further, the sample included both male and female participants, and while we controlled for the effect of sex within our mixed models, we were underpowered to perform any subgroup analysis. Future work is needed to explore if patient demographics (e.g., sex), surgical procedures (e.g., graft type), and specific rehabilitation strategies affect muscle structure and function following ACLR. Finally, it is important to recognize T2 relaxation time changes in response to exercise are highly individual and can be influenced by factors such as the metabolic capacity and vascular dynamics of the active tissue [18].

## 6 Perspective

The findings from this study highlight deficits in lower limb muscle size and activity following ACLR that persist even after seemingly successful rehabilitation and the return to pre‐injury levels of training and competition. Notably, quadriceps atrophy was associated with altered muscle activity and asymmetrical jump strategy, suggesting that targeted interventions to restore quadriceps size may improve knee joint function and performance. While several exercise strategies have shown promise in improving quadriceps muscle size in the reconstructed limb [37], the optimal strategies to redress quadriceps atrophy following ACLR remain unknown [38, 39]. Similarly, rehabilitation of the semitendinosus following tendon harvest remains a challenge, as exercises that preferentially target semitendinosus in uninjured limbs, such as the Nordic hamstring curl [40, 41], are less effective at recruiting this muscle post‐ACLR [21]. As a consequence, it may be advantageous to develop rehabilitation strategies that specifically target the semimembranosus—the only remaining internal rotator of the knee that is also a hip extensor. For example, hip extension training stimulates significantly more semimembranosus hypertrophy than knee dominant exercises [40]. Finally, the role of the triceps surae (soleus and gastrocnemius) in post‐ACLR recovery remains unexplored. Further research is needed to determine if deficits in the structure and function of the lower limb muscles following ACLR influence performance during high‐speed, athletic tasks, and risk of secondary injury.

## Conflicts of Interest

Associate Professor Matthew N. Bourne is supported by an Advance Queensland Industry Research Fellowship in partnership with VALD. The authors have no other competing interests to declare that are relevant to the content of this article.

## Data Availability

The data that support the findings of this study are available on request from the corresponding author. The data are not publicly available due to privacy or ethical restrictions.
